# High Inorganic Triphosphatase Activities in Bacteria and Mammalian Cells: Identification of the Enzymes Involved

**DOI:** 10.1371/journal.pone.0043879

**Published:** 2012-09-12

**Authors:** Gregory Kohn, David Delvaux, Bernard Lakaye, Anne-Catherine Servais, Georges Scholer, Marianne Fillet, Benjamin Elias, Jean-Michel Derochette, Jacques Crommen, Pierre Wins, Lucien Bettendorff

**Affiliations:** 1 GIGA-Neurosciences, University of Liège, Liège, Belgium; 2 Laboratory of Analytical Pharmaceutical Chemistry, Department of Pharmaceutical Sciences, CIRM, University of Liège, Liège, Belgium; 3 Institute of Condensed Matter and Nanosciences, Université catholique de Louvain, Louvain-la-Neuve, Belgium; 4 Prayon S.A., Engis, Belgium; New England Biolabs, Inc., United States of America

## Abstract

**Background:**

We recently characterized a specific inorganic triphosphatase (PPPase) from *Nitrosomonas europaea*. This enzyme belongs to the CYTH superfamily of proteins. Many bacterial members of this family are annotated as predicted adenylate cyclases, because one of the founding members is CyaB adenylate cyclase from *A. hydrophila*. The aim of the present study is to determine whether other members of the CYTH protein family also have a PPPase activity, if there are PPPase activities in animal tissues and what enzymes are responsible for these activities.

**Methodology/Principal Findings:**

Recombinant enzymes were expressed and purified as GST- or His-tagged fusion proteins and the enzyme activities were determined by measuring the release of inorganic phosphate. We show that the hitherto uncharacterized *E. coli* CYTH protein ygiF is a specific PPPase, but it contributes only marginally to the total PPPase activity in this organism, where the main enzyme responsible for hydrolysis of inorganic triphosphate (PPP_i_) is inorganic pyrophosphatase. We further show that CyaB hydrolyzes PPP_i_ but this activity is low compared to its adenylate cyclase activity. Finally we demonstrate a high PPPase activity in mammalian and quail tissue, particularly in the brain. We show that this activity is mainly due to Prune, an exopolyphosphatase overexpressed in metastatic tumors where it promotes cell motility.

**Conclusions and General Significance:**

We show for the first time that PPPase activities are widespread in bacteria and animals. We identified the enzymes responsible for these activities but we were unable to detect significant amounts of PPP_i_ in *E. coli* or brain extracts using ion chromatography and capillary electrophoresis. The role of these enzymes may be to hydrolyze PPP_i_, which could be cytotoxic because of its high affinity for Ca^2+^, thereby interfering with Ca^2+^ signaling.

## Introduction

Recently, we have demonstrated for the first time that a bacterial enzyme hydrolyzed inorganic triphosphate (tripolyphosphate, PPP_i_) with high specificity and catalytic efficiency [1]. This enzyme from *Nitrosomonas europaea* (referred to as *Neu*TTM) belongs to the CYTH protein superfamily identified in 2002 by Iyer and Aravind [2]. According to these authors the catalytic domains of human 25-kDa thiamine triphosphatase (hThTPase, [3]) and CyaB-like adenylyl cyclase from *Aeromonas hydrophila* (AC2, [4]) define a novel superfamily of domains supposed to bind “organic phosphates”. This superfamily was therefore called “CYTH” (CyaB-THiamine triphosphatase), and the presence of orthologs was demonstrated in all three superkingdoms of life. Using multiple alignments and secondary structure predictions, Iyer and Aravind showed that the catalytic core of CYTH enzymes contained a novel α+β scaffold with 6 conserved acidic and 4 basic residues. At least 4 of the acidic residues (generally glutamates) are likely to chelate 1 or 2 divalent cations that are required for catalysis, as is the case for nucleotide cyclases, polymerases and some phosphohydrolases [2], [5], [6]. Independently of those bioinformatic studies, Shuman and coworkers investigated the first step of RNA capping in fungi and protozoa catalyzed by RNA triphosphatases. They showed that the yeast RNA triphosphatase Cet1 belongs to a family of metal-dependent phosphohydrolases, whose active site is located within a topologically closed hydrophilic β-barrel (composed of 8 antiparallel β strands) that they called the “triphosphate tunnel” [7]. Strikingly similar structures were found for several bacterial and archaeal proteins of unknown function [7]. Since all those proteins carry the CYTH signature, Gong et al., [7] coined the name “Triphosphate Tunnel Metalloenzyme” (TTM) for a superfamily including Cet-1-like RNA triphosphatases and all other CYTH proteins. However, the *Neu*TTM PPPase does not exhibit the closed tunnel structure [1]. On the other hand, bacterial CYTH enzymes have generally been annotated as adenylyl cyclases, but only AC2 from *A. hydrophila* [4] and YpAC4 form *Y. pestis* [8] have been found to exhibit adenylyl cyclase activity, and its physiological significance in these organisms is unclear.

Only two other bacterial CYTH enzymes have been functionally characterized: *Neu*TTM [1] and *Cth*TTM from *Clostridium thermocellum* [9], [10]. Both have a high PPPase activity (though *Cth*TM was less specific) but neither had any significant adenylyl cyclase activity [1], [9]. We thus suspect that PPPase activity might be the ancestral role of CYTH proteins, but the significance of such an activity is by no means clear. Indeed, in contrast to high molecular weight polyphosphates, no data are available concerning the presence or the possible role of PPP_i_ (and other short chain polyphosphates) in either prokaryotic or eukaryotic cells. However, this lack of data is essentially due to the current absence of sensitive and specific methods to detect short chain polyphosphates. Actually, it is not excluded that these compounds might play very important roles in cell physiology.

The aim of the present investigation was to check whether significant PPPase activities exist in *E. coli* but also in mammalian tissues and what enzymes are responsible for these activities.

## Results

### Characterization of recombinant *E. coli* ygiF protein

**Figure 1:**
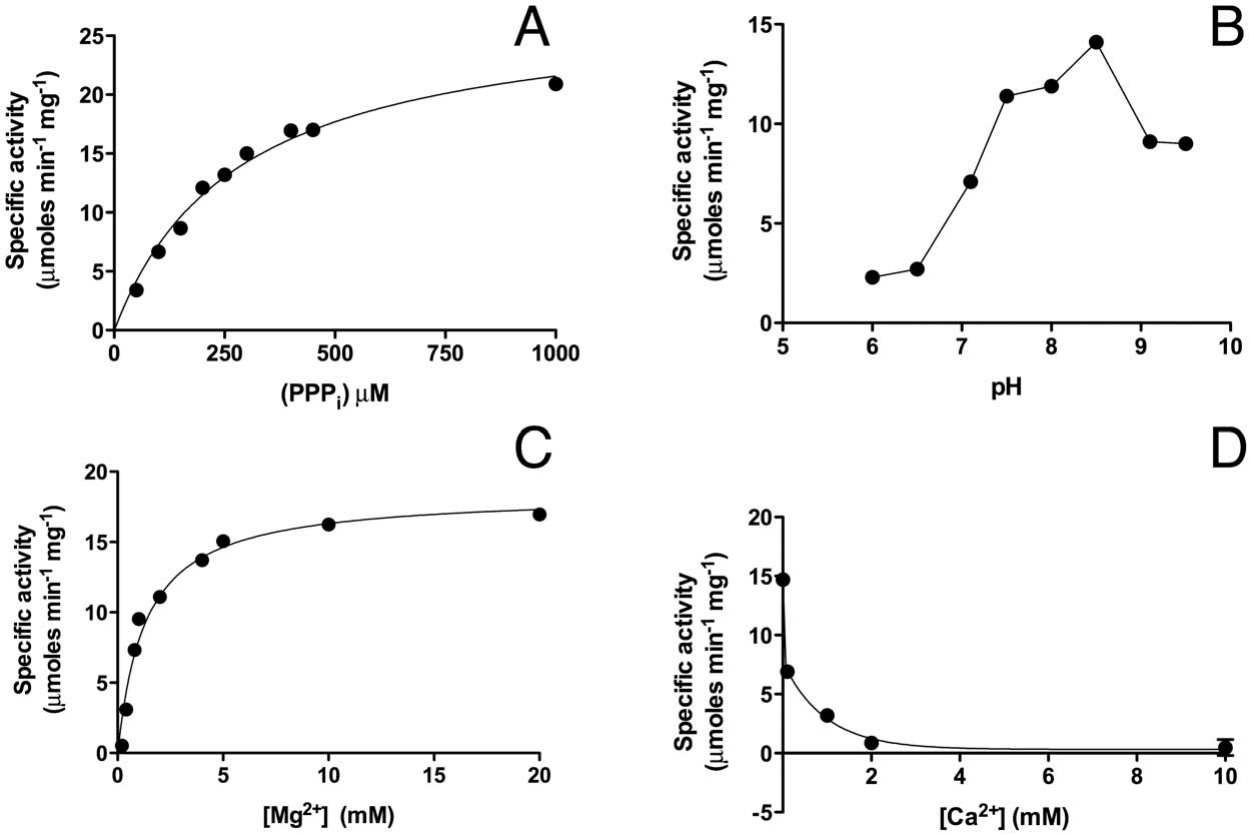
Enzymatic characterization of GST-ygiF PPPase activity. (A) Enzyme activity as a function of PPP_i_ concentration. The curve was obtained by non-linear fitting to the Michaelis-Menten equation. The Mg^2+^ concentration was 5 mM. (B) PPPase activity as a function of pH at 0.5 mM Mg^2+^. (C) PPPase activity as a function of Mg^2+^ concentration. (D) Inhibition of PPPase activity by Ca^2+^ in the presence of Mg^2+^ (5 mM). If not otherwise stated, the substrate concentration was 0.5 mM and the incubation was carried out at 50°C and at pH 8.5. (Means ± SD, n = 3).

**Figure 2:**
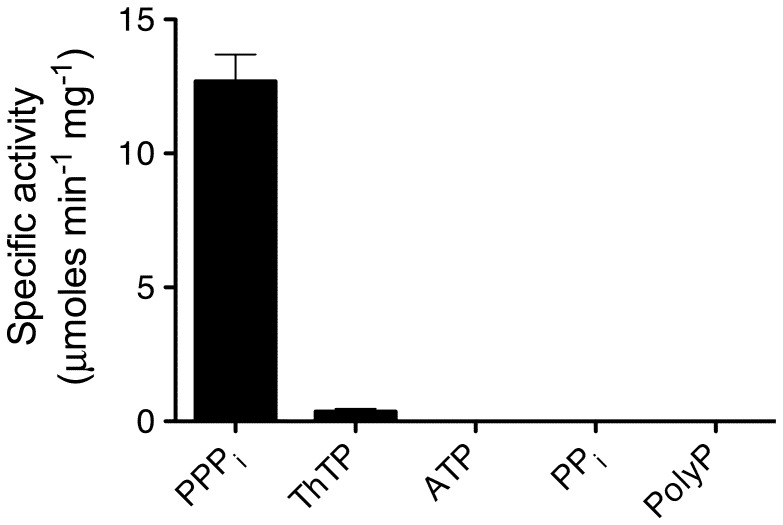
Specificity of GST-ygiF. The various substrates were tested at a concentration of 0.5 mM in the presence of 5 mM Mg^2+^. The incubation temperature was 50°C and the pH 8.5. (Means ± SD, n = 3).

As we have previously shown that the *N. europaea* CYTH protein is a specific tripolyphosphatase [1], we wanted to check whether the *E. coli* ortholog ygiF [2], labeled as a predicted adenylate cyclase, had a similar activity and substrate specificity. YgiF was overexpressed in *E. coli* as a GST-fusion protein and purified (**Figure S1**). The purified fusion protein had indeed a high PPPase activity (**Figure 1**) with a *V*_max_ of 27±2 µmoles min^−1^ mg^−1^ and the *K*_m_ was 270±25 µM. The optimum pH was 8.5. The enzyme was activated by Mg^2+^ (EC_50_ = 1.3±0.2 mM) and inhibited by Ca^2+^ (IC_50_ = 0.6±0.3 mM). Among other substrates tested, only ThTP was slowly hydrolyzed. No hydrolysis was observed with ATP, ITP, GTP, PP_i_ or polyphosphate (65±5 residues) as substrates (**Figure 2**).

These results suggest that ygiF, like *Neu*TTM, is a specific tripolyphosphatase, with the characteristic properties of the CYTH protein family, *i. e.* alkaline optimum pH, activation by Mg^2+^ and inhibition by Ca^2+^.

### In *E. coli* supernatants, hydrolysis of PPP_i_ is mainly catalyzed by inorganic pyrophosphatase

We first estimated the PPPase activity of a crude extract from wild-type MG1655 E. coli (supernatant obtained after sonication and centrifugation at 40,000×g). We found a relatively high Mg^2+^-dependent PPPase activity with alkaline pH optimum: the specific activity was about 0.2 µmol min^−1^ mg^−1^ at 37°C (with [PPP_i_] = 1 mM, [Mg^2+^] = 5 mM, pH 9.1). The activity was remarkably heat-stable (up to 80°C).

Those properties are reminiscent of *E. coli* inorganic pyrophosphatase (EcPPase) [11], [12]. We therefore suspected that PPP_i_ hydrolysis by *E. coli* extracts might be mainly catalyzed by the PPase, which is abundant in the bacterial cytoplasm [13].

We partially purified the enzyme responsible for the high PPPase activity in *E. coli* supernatants using various chromatographic techniques (**Table S1**), followed by native electrophoresis on agarose gels and in-gel activity determination. The band containing the activity was then excised. Analysis by LC-ESI/MS/MS identified inorganic pyrophosphatase (PPase) as a major protein present in the fraction.

**Figure 3:**
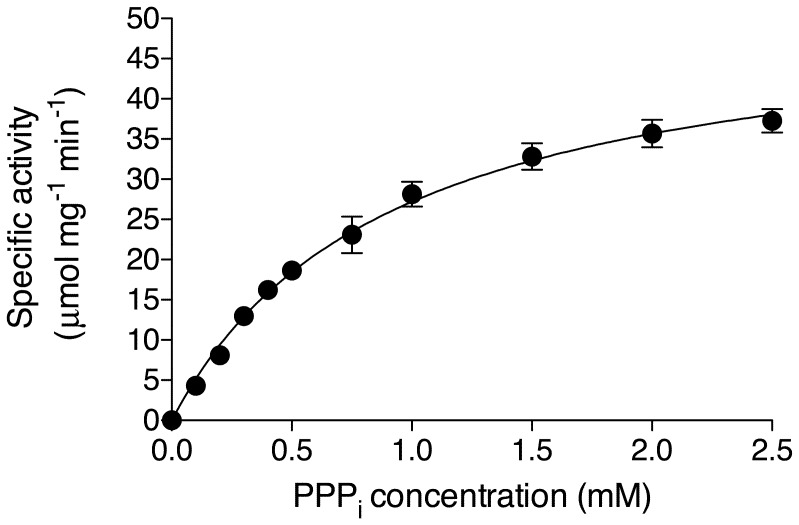
Enzyme activity of commercial *E. coli* recombinant pyrophosphatase as a function of PPP_i_ concentration. The enzyme was incubated in the presence of 10 mM Mg^2+^ in 50 mM CHES buffer at pH 9.1, for 30 min at 25°C. (Means ± SD, n = 3).

We therefore used a commercially available preparation of purified EcPPase to characterize its PPPase activity (**Figure 3**). This preparation indeed hydrolyzed PPP_i_ under the conditions observed above. As expected, it was less efficient for the hydrolysis of PPP_i_ than for PP_i_, considered its natural substrate. With PPP_i_, we found a *V*_max_ = 50 µmol min^−1^ mg^−1^ at 25°C about 40% of *V_max_* measured with PP_i_ under similar conditions (*V*_max_ = 125 µmol min^−1^ mg^−1^). The *K_m_* for PPP_i_ was 0.91±0.09 mM, three orders of magnitude higher than for PP_i_ (≤10^−3^ M compared to ≤10^−6^ M) [14].

Considering a molecular mass of 20 kDa per subunit, we estimated that *k_cat_* = 16.7 s^−1^ at 25°C for PPP_i_, compared to 42 s^−1^ for PP_i_. Using a different preparation of recombinant *E. coli* pyrophosphatase, Avaeva *et al.* [14] reported a substantially higher value for *k*_cat_ (389 s^−1^ with PP_i_). In any event, due to the very large difference in *K*_m_ values for PP_i_ and PPP_i_, the catalytic efficiency (*k*_cat_/*K*_m_) is much lower for PPP_i_ (18.10^3^ s M^−1^) than for PP_i_ (≥42.10^6^ s M^−1^).Considering that the intracellular PP_i_ concentration is relatively high, except under severe energy stress [13], it is likely that most of the active sites of pyrophosphatase remain saturated with PP_i_ and unavailable for PPP_i_ hydrolysis. In addition, the catalytic efficiency for PPP_i_ hydrolysis is low. Therefore, it may be argued that, *in vivo*, the pyrophosphatase is uneffective as a PPPase and ygiF, which is much more specific, might be the main enzyme responsible for PPP_i_ hydrolysis *in vivo*. We thus estimated the PPPase activity in the ygiF knockout strain JW 3026-2. No decrease of specific PPPase activity in crude extracts was observed (**Figure S2**), indicating that the contribution of ygiF to PPPase activity is, at best, marginal. This is not surprising as PPase is highly expressed in *E. coli* with nearly 400 copies per cell [15]. On the other hand, ygiF is expressed at only 20 copies per cell. It is therefore not surprising that ygif does not contribute significantly to PPP_i_ hydrolysis in a bacterial supernatant fraction. For ygiF to play a significant role in PPP_i_ hydrolysis, it would require at least a 20-fold upregulation of its expression. This is however not very plausible as it is constitutively cotranscribed with glutamine synthetase adenylyl transferase [16]. Also in contrast to inorganic pyrophosphatase, ygiF is not an essential protein.

As *E. coli* also contains an exopolyphosphatase which might be responsible for some PPP_i_ hydrolysis, we used a mutant strain devoid of both polyphosphate kinase and exopolyphosphatase (Δ*ppk-ppx::km*). However, PPPase activity was the same in this strain as in the wild-type MG1655 strain (**Figure S3**), suggesting that exopolyphosphatase does not contribute significantly to PPP_i_ hydrolysis in an *E. coli* supernatant, in agreement with previous results that showed that PPP_i_ is not a good substrate for this enzyme [17].

We also tested whether PPase and PPPase activity changed during growth of *E. coli*, but both PPase and PPPase activities remained relatively constant (**Figure S4**).

### CyaB adenylate cyclase has a significant triphosphatase activity but lacks specificity

**Figure 4:**
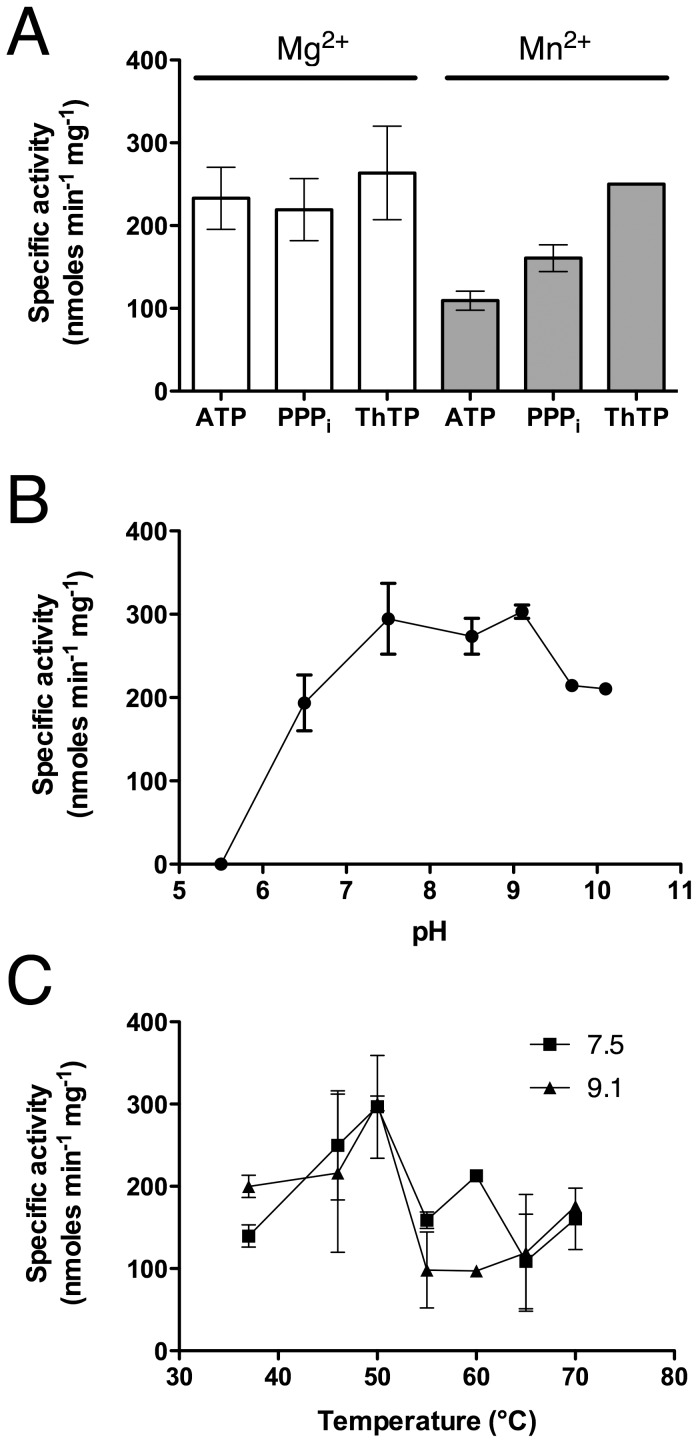
Enzymatic hydrolysis of ATP, PPP_i_ and ThTP by recombinant CyaB protein. (A) Specificity of CyaB in the presence of 5 mM Mg^2+^ or Mn^2+^ at pH 9.1. (B) pH dependence of ThTPase activity. (C) Temperature dependence of ThTPase activity at pH 7.5 and 9.1. If not otherwise stated, the substrate concentration was 0.5 mM, the Mg^2+^ or Mn^2+^ concentration was 5 mM and a temperature of 50°C was used. (Means ± SD, n = 3).

The *A. hydrophila* CyaB protein, one of the founding members of the CYTH protein superfamily has an adenylate cyclase activity, but is different from class 1 adenylyl cyclases present in most organisms [4]. Furthermore, CyaB was not expressed under normal laboratory growth conditions. As for ygiF, we wanted to test whether this enzyme is able to hydrolyze triphosphates. Our results show that it indeed hydrolyzes substrates such as ATP, ThTP and PPP_i_ but at a low rate (**Figure 4A**). In the presence of Mg^2+^ it has no preference for any of these substrates, while it has a slight preference for ThTP over ATP and PPP_i_ in the presence of Mn^2+^. The rate of hydrolysis of these substrates is at least an order of magnitude lower than its adenylate cyclase activity previously reported [4] and respectively 2 and 3 orders of magnitude lower than the PPPase activity of ygiF (this study) and *Neu*TTM [1]. Therefore, this activity is probably not of physiological importance. With ThTP as substrate, the optimum pH is alkaline (**Figure 4B**) as is usually observed with enzymes of the CYTH superfamily and the activity is highest at a temperature of 50°C (**Figure 4C**).

### PPPase activities in mammalian tissues

**Figure 5:**
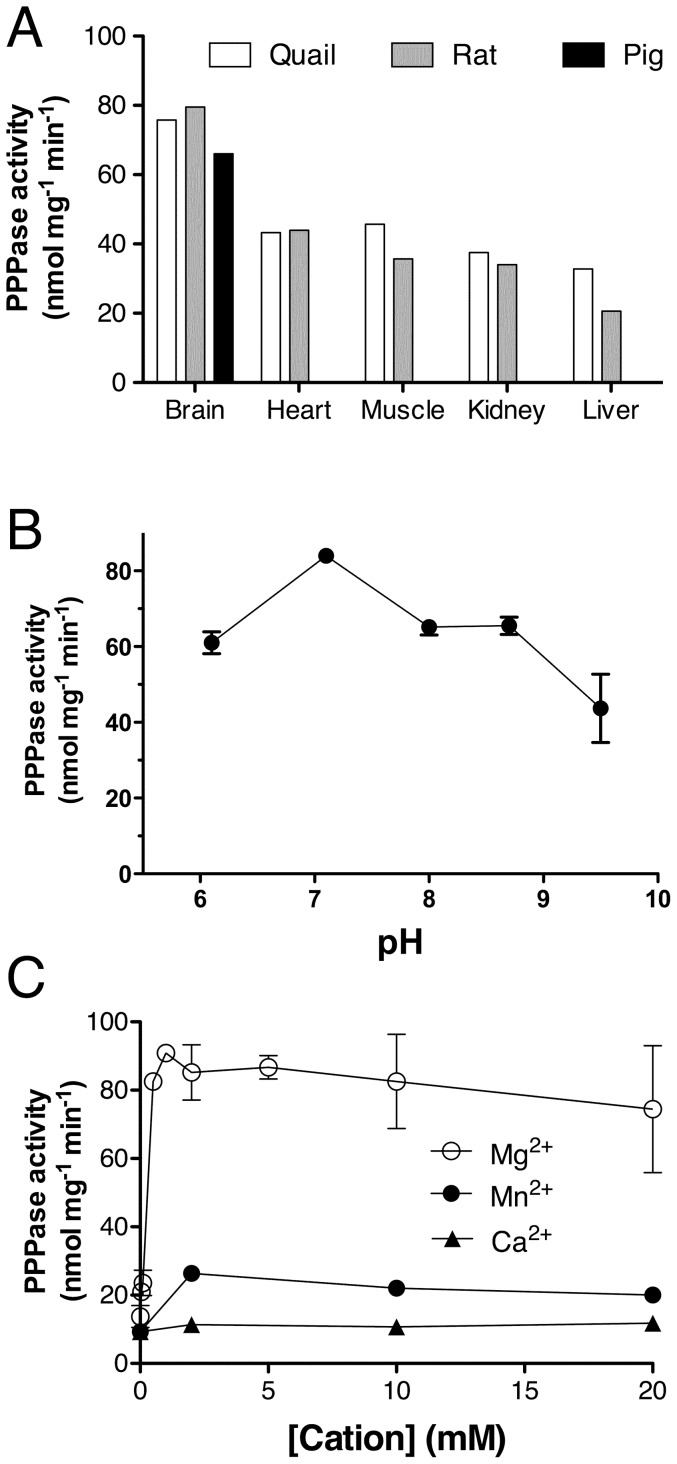
PPPase activities in animal tissue supernatant fractions. (A) PPP_i_ hydrolysis in various organs of the rat, and the quail, and in pig brain at pH 7.0 in the presence of 5 mM Mg^2+^ (n = 3–9). (B) pH dependence of PPPase hydrolysis in rat brain in the presence of 5 mM Mg^2+^ (n = 3). (C) Activation of PPPase activity by divalent cations. The PPP_i_ concentration was 0.5 mM and the incubation was carried out at 37°C. (Means ± SD, n = 3–9).

As the above results suggest that PPPase activities are rather common in bacteria, we wanted to test whether such activities are also present in mammalian tissues. We measured PPP_i_ hydrolysis in the supernatant fraction of several rat and quail tissues as well as pig brain (**Figure 5A**). The specific PPPase activity was highest in brain, only 2–3 times lower than in *E. coli* supernatants under identical conditions. The activity was not strongly pH-dependent but was highest around pH 7 (**Figure 5B**). PPPase activity was activated by Mg^2+^, to a lesser extent by Mn^2+^, but was insensitive to Ca^2+^ (**Figure 5C**).

In order to identify the enzyme responsible for PPPase hydrolysis in mammalian brain, we used a combination of chromatographic techniques and native polyacrylamide gel electrophoresis, and we carried out the identification by LC-ESI/MS/MS. A major protein identified was the pig homologue of the *Drosophila* protein prune. It was recently shown that human prune is an exopolyphosphatase with higher activity towards short chain polyphosphates such as PPP_i_ and P_4_ [18]. It also hydrolyzes very efficiently adenosine and guanosine tetraphosphates. For PPP_i_, the *k*_cat_ was 13 s^−1^ and the *K*_m_ was as low as 2.2 µM. This suggest that most of the PPPase activity in mammalian tissues is due to h-Prune. The latter belongs to the DHH protein superfamily named after the characteristic N-terminal Asp-His-His motif. It has no sequence similarities with proteins of the CYTH superfamily (**Figure S5**).

h-Prune is often overexpressed in metastatic cancer and interacts with the metastasis tumor suppressor nm23-H1, coding for nucleoside diphosphate kinase A (NDPK-A). It was shown that phosphorylation of nm23-H1 by casein kinase 1 leads to complex formation with h-Prune, hence promoting cell motility in breast cancer cells. The role of h-Prune in normal adult brain cells is unknown. H-Prune and nm23-H1 are co-expressed during embryonic development and may play a role in mammalian brain development, while in the adult brain their role may be predominantly linked to their respective enzyme activities [19].

### Is PPP_i_ present in living cells?

**Figure 6:**
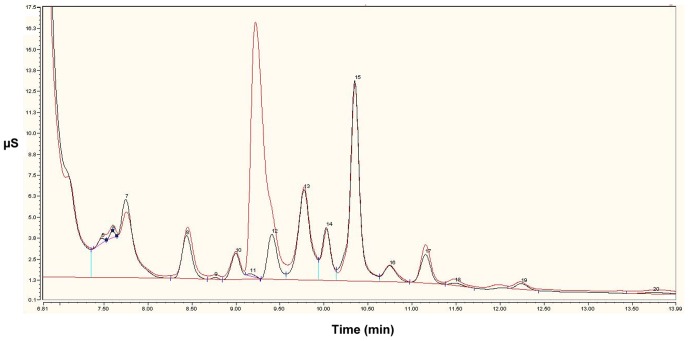
Separation of a *E. coli* extract on a Dionex column. The sample was spiked with exogenous PPP_i_ (10 mg/L). It can be estimated that peak 11 accounts for less than 0.5 mg/L.

Until now PPP_i_ has not been shown to exist in living cells except for very specialized organelles such as protozoan acidocalcisomes [20]. One of the problems when determining PPP_i_ is the absence of a specific and sensitive detection method. We first tested a chromatographic separation technique using a Dionex device equipped with a conductivity detector. Such a method was already used to detect exogenously added tripolyphosphate in frozen cod and scallop adductor [21]. We were able to detect in *E. coli* a small peak at the retention time of PPP_i_ corresponding to a concentration of 1 µM, while no peak was detected in rat brain extracts (**Figure 6**).

**Figure 7:**
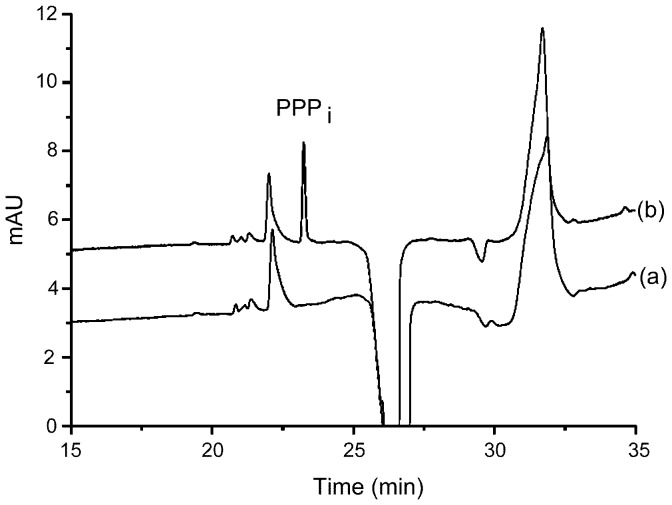
Separation of PPP_i_ by capillary electrophoresis. Electropherograms of (a) *E. coli* supernatant after fractionation on G-25 column and (b) *E. coli* supernatant after fractionation on G-25 column spiked with 3 µM PPP_i_.

A method using capillary electrophoresis (CE) coupled to a diode array detector was also optimized to detect PPP_i_ with a limit of detection estimated to 0.5 µM. After fractionation of *E. coli* supernatants on G-25 column, no PPP_i_ peak could be observed (**Figure 7**), suggesting that PPP_i_, if it exists in living cells, is not a very abundant compound and its concentrations are probably in the low micromolar or even sub-micromolar range.

## Discussion

Next to carbon, oxygen, nitrogen and hydrogen, phosphorus is the most abundant element in living organisms. Phosphorus is found mainly under the form of phosphate in many organic compounds such as nucleotides, metabolic intermediates (intermediates of glycolysis such as phosphoenolpyruvate for instance), phospholipids or in bones as calcium phosphate salts. In addition to be present in organic molecules, phosphates may exist in free forms. Hydrolysis of ATP yields P_i_ whose intracellular concentration may exceed 1 mM. P_i_ is recycled mainly by regeneration of ATP via F_o_F_1_-ATP synthase according to the reaction ADP+P_i_ 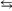
ATP.

Inorganic pyrophosphate (PP_i_) is released in different anabolic processes such as the synthesis of ribo- and deoxyribonucleotides ((NMP)_n_+NTP
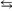
(NMP)_n+1_+PP_i_) or the synthesis of aminoacyl-tRNA (amino acid+tRNA+ATP
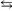
aminoacyl-tRNA+AMP+PP_i_). In each case, PP_i_ is rapidly hydrolyzed to 2 P_i_ by an inorganic PPase (EC 3.6.1.1), rendering these reactions globally irreversible. PPases are widely distributed in all organisms and here, we show that at least *E. coli* PPase also has an important PPPase activity. Hence, PP_i_ is constantly formed in all organisms, but it is not synthesized *de novo*, at least in animal cells [22], though a pyrophosphate synthase, related to V-type ATPases, has been characterized from *Rhodospirillum rubrum* [23].

Long chain polyphosphates have been discovered in many organisms [24], [25]. These polyphosphates are linear chains of up to 1000 P_i_ residues linked by phosphoanhydride bonds. They may play multiple roles as energy sources, phosphate reservoirs, phosphate donors and chelators of divalent cations. In *E. coli*, they are synthesized according to the reaction (polyP)_n_+ATP
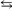
(polyP)_n+1_+ADP. A recent study suggested that polyP could be synthesized in mammalian cells by a mechanism requiring F_o_F_1_-ATP synthase [26]. Polyphosphates may be hydrolyzed by dedicated exopolyphosphatases, but less specific enzymes such as *Clostridium thermocellum* CYTH protein [10] or h-Prune for instance [18] may also act as exopolyphosphatases.

PPP_i_ has never been demonstrated to exist in living organisms with the exception of the very specialized protozoan acidocalcisomes [20]. PPP_i_ can be generated as the final product of polyP-glucose phosphotransferase (EC 2.7.1.63), present in various bacteria (*Mycobacterium tuberculosis*, *Propionobacterium shermanii*…). This enzyme uses polyP to form glucose 6-phosphate from glucose ((polyP)_n_+D-glucose???(polyP)_n−1_+D-glucose 6-phosphate). In animals, an endopolyphosphatase (EC 3.6.1.10) may produce PPP_i_ [27]. PPP_i_ may also serve as a substrate for the phosphorylation of membrane proteins in rat liver microsomes *in vitro* [28]. This activity is probably not of physiological significance as it is inhibited by micromolar concentrations of ADP and ATP.

The observation that *Neu*TTM is a highly specific PPPase raises the question of the existence of this compound in living cells [1]. Moreover, our present results show that ygiF, which is commonly annotated a predicted adenylate cyclase, is also a relatively specific PPPase. Its activity is however insignificant compared to PPPase activity resulting from inorganic PPase in *E. coli*.

**Figure 8:**
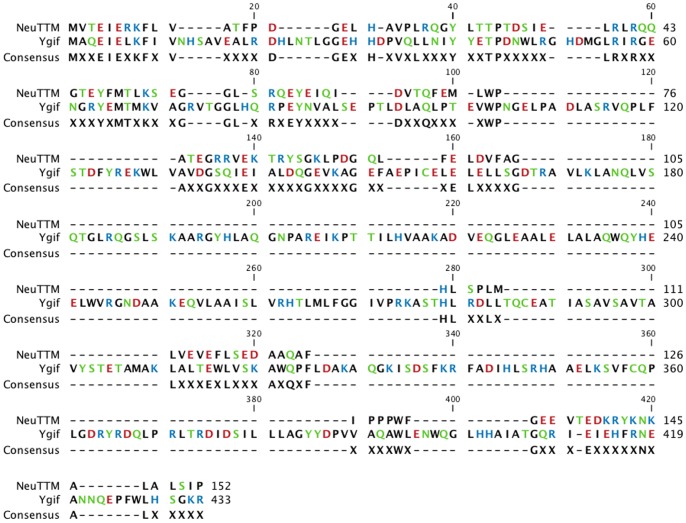
Sequence alignment of *Neu*TTM and ygiF. The sequence alignment was made using CLC Sequence Viewer 6 (CLC bio A/S, 8200 Aarhus, Denmark).

**Figure 8** shows the comparison of the sequences of *Neu*TTM and ygiF. It is obvious that ygiF is about twice as long as *Neu*TTM. We previously suggested that the catalytic dyad in *Neu*TTM is formed by Tyr-28 and Lys-52. Though the equivalent of Tyr-28 is present in ygiF, the equivalent of Lys-52 seems to be absent. Furthermore, the specific activity of ygiF is one or two orders of magnitude lower than that of *Neu*TTM. These results suggest that the physiological role of ygiF might be different from PPP_i_ hydrolysis, but it is probably not an adenylate cyclase, as we were unable to detect such activity in purified ygiF.

We also studied another protein from the CYTH protein family: the *A. hydrophyla* CyaB protein, which was previously shown to have a significant adenylate cyclase activity. Our data show that CyaB also has PPPase activity, but this activity is lower than its adenylate cyclase activity [4]. Finally, we show for the first time a high PPPase activity in animal tissues, and in particular in brain. This PPPase activity is catalyzed by the homologs of the *Drosophila* exopolyphosphatase prune.

Our results show a high PPPase activity in many cells from bacteria to birds and mammals. However, few of these activities come from specific PPPases: the only known specific PPPases are *Neu*TTM and ygiF, though it is questionable that for the latter, this activity is of physiological importance.

In view of the many and important PPPase activities present in cells, the issue is to know whether PPP_i_ exists as such in living cells. This compound is difficult to detect in small amounts: there are no specific enzymatic assays and it does not contain any chromophore or fluorophore. We first tested an anion exchange separation method coupled to electrochemical detection. This method is routinely used to test the composition of industrial preparations of small-chain polyphosphates. A small peak with a retention time corresponding to PPP_i_ was detected in *E. coli* but not in brain extracts. We then developed a method based on a separation of phosphates by capillary electrophoresis. Again, no definite conclusion could be drawn as to the existence of PPP_i_ in living cells. However, our data allow us to draw the conclusion that if it exists, intracellular concentrations hardly exceed 1 µM. This would be in agreement with the high PPPase activities observed. Furthermore, many PPP_i_ hydrolyzing enzymes are proteins that are constitutively expressed with a relatively constant expression (inorganic pyrophosphatase, mammalian prune). It is therefore not likely that intracellular PPP_i_ concentrations undergo large variations, as it would be the case for a signaling molecule for instance.

PPP_i_ has a very strong chelating power for divalent cations and in particular Ca^2+^, which is the reason why it was extensively used in detergents to control water hardness. The dissociation constant *K*_d_ for Ca^2+^ is approximately 10^−7^ M [29]. This would mean that if it accumulated in cells, it would be expected to interfere with Ca^2+^ signaling. It is therefore possible that the existence of PPPase activities of widely expressed less specific phosphohydrolases is a protective means to prevent accumulation of intracellular PPP_i_ that might accumulate from degradation of long chain polyphosphates for instance. From this point of view, specific PPPases such as *Neu*TTM could thus be considered as metabolite proofreading enzymes, an expression recently coined by Van Schaftingen and colleagues, by analogy with the proofreading enzymes involved in DNA repair for instance [30], [31], and which should protect cells from unwanted toxic metabolic side-products.

On the other hand, it cannot be excluded that PPP_i_, which is an energy-rich compound and the simplest triphosphate that can be imagined, may have played a role in energy metabolism in the earliest organisms. Therefore, we recently suggested that PPP_i_ hydrolysis could be the primitive activity of the CYTH protein family [1]. But with the appearance of PPPase side-activity in other abundant phosphohydrolases, CYTH proteins could have evolved towards more complex activities, such as adenylate cyclase in *A. hydrophila* [4], mRNA–triphosphatase in *S. cerevisiae* and some protozoans [7], [32], [33] or thiamine triphosphatase in mammals [3], [34].

## Materials and Methods

### Materials

Sodium triphosphate (PPP_i_), sodium trimetaphosphate (Cyclic PPP_i_), polyphosphate (sodium phosphate glass, 65±5 residues), ATP, ITP, GTP, guanosine 5′- tetraphosphate (Gp_4_, tris salt) and *E. coli* pyrophosphatase were from Sigma-Aldrich NV/SA (Bornem, Belgium). Thiamine triphosphate (ThTP) was synthesized and purified as previously described [35]. The CyaB expression plasmid pTRC99ACyaB was a gift of Drs Antoine Danchin and Agnieszka Sekowska (AMAbiotics SAS, Genopole Campus 1 - Genavenir 8, 5, rue Henri Desbruères, 91030 EVRY Cedex, France). All animal experiments were made in accordance with the directives of the committee for animal care and use of the University of Liège and in accordance with the European Communities Council Directive of November 24, 1986 (86/609/EEC). The protocols were approved by the Committee on the Ethics of Animal Experiments of the University of Liège (# 823 for rats and # 727 for quails). The animals were killed by decapitation and all efforts were made to minimize suffering. Pig brains were obtained from the local slaughterhouse (Abattoirs Publics de Liège & Waremme SC, rue de Droixhe 15, 4020 Liège, Belgium) with the permission to be used for experimental purposes at the University of Liège.

### Bacterial strains

JW3026-2 (F-, Δ(araD-araB)567, ΔlacZ4787(::rrnB-3), λ^−^, ΔygiF747::kan, rph-1, Δ(rhaD-rhaB)568, hsdR514, CGSC#10312) was from the *E. coli* Genetic Stock Center (Yale University, New Haven CT, USA). The CF5802 strain lacking polyP kinase (PPK) and exopolyphosphatase (Δ*ppk-ppx::km*) [36] was a gift from Dr. M. Cashel (Laboratory of Molecular Genetics, NICHD, National Institutes of Health, Bethesda, MA).

### Determination of phosphohydrolase activities

The standard incubation medium contained 50 mM buffer, 5 mM MgCl_2_, 0.5 mM PPP_i_ and 20 µL of the enzyme at the adequate concentration in a total volume of 100 µL. The mixture was incubated at 37 or 50°C, the reaction was stopped by addition of 1 mL phosphate reagent [37] and the absorbance (UV-1800 Shimadzu UV spectrophotometer) was read at 635 nm after 30 min and compared with a standard curve (0–0.5 mM K_2_HPO_4_) to estimate the release of inorganic phosphate. The buffers used for incubation at different pH values were: Na-MOPS (pH 7.0–7.5), Na-HEPES (pH 7.5–8.0), Na-TAPS (pH 8.0–9.0), Na-CHES (pH 9.0–10.0) and Na-CAPS (pH 10.0–10.5).

### Purification of PPPase activity from *E. coli* supernatants

*E. coli* wild type strain MG1655 was grown overnight in LB medium at 37°C under agitation (250 rpm). The bacteria were harvested by centrifugation for 10 minutes, at 8000×g at 4°C. The pellet was suspended in Hepes-buffer (30 mM Hepes, 50 mM NaCl, pH 7.2–7.4) and cells were lysed by high pressure using a French press. The lysate was centrifuged for 30 minutes at 40,000×g at 18°C. The supernatant (called S1) was brought to 50% saturation with ammonium sulfate and kept under constant stirring for one hour at room temperature. After centrifugation for 30 minutes at 20,000×g at 18°C, the supernatant (called S50) was brought to 80% saturation with ammonium sulfate and kept under constant stirring for one hour at room temperature. After centrifugation 30 minutes at 20,000×g at 18°C, the pellet was suspended in Hepes-buffer. The preparation obtained (called P80) was loaded on a monoQ column coupled to a Fast Protein Liquid Chromatography (FPLC) apparatus and elution was carried out with a linear NaCl gradient from 50 to 500 mM. The fractions with the highest PPPase activity were pooled (called F1) and placed on a Sephadex G200 column (1×25 cm) equilibrated with Hepes-buffer. Elution was performed in the same buffer. The fractions with the highest PPPase activity were pooled (called F2) and used to perform a non-denaturing polyacrylamide gel separation and the PPPase activity was determined as described below. The bands containing PPPase activity were cut and analyzed by liquid chromatography coupled on-line to positive mode electrospray ionization tandem mass spectrometry (UltiMate 3000 Nano LC Systems, Dionex, AmaZone, Bruker) for protein identification.

### In-gel enzyme activities

Non-denaturating polyacrylamide gels (10%) were run in Tris-Glycine buffer (25 mM Tris, 190 mM glycine). After incubation for 30 minutes in a bath containing PPP_i_ (0.5 mM), buffer (20 mM), and MgCl_2_ (1 mM), the gel was colored with phosphate precipitation reagent (1% v/v ammonium heptamolybdate, 1% v/v triethylamine, 1 N HNO_3_) [38]. For *E. coli* PPPase, the incubation temperature was 50°C and buffer was Na-CHES (pH 9.5) while for pig brain PPPase incubation temperature was 37°C and buffer was Na-MOPS (pH 7.1).

### Overexpression and purification of recombinant ygiF

YgiF was produced in *E. coli* as a GST fusion protein, as previously described [39]. Genomic DNA was isolated from *E. coli* DH5α bacteria using a standard protocol (Invitrogen). YgiF coding sequence was amplified from 1 µg of genomic DNA using Pfu polymerase and 35 PCR cycles of denaturation (94°C, 20 s), annealing (64°C, 30 s) and elongation (72°C, 90 s) using forward (5′-TTTGGATCCATGGCTCAGGAAATCGAATTAAAG-3′) and reverse (5′-TTTGCGGCCGCTTAACGTTTTCCGCTGTGCAACC-3′) primers. The fragment was then incubated for 15 minutes at 72°C in the presence of Taq polymerase and dATP to add a polyA tail. The 1.3 kb PCR fragment was cloned by TA cloning in the pGEM-T vector (Promega). The plasmid containing the insert was sequenced as described above and then digested by BamHI and NotI. The released fragment was purified on agarose gel and subcloned into pGEX-4T2 (GE Healthcare) to produce the GST-ygiF fusion protein in *E. coli* DH5α. The GST-fusion protein was purified using a 1 mL GSTrap HP column (GE Healthcare).

### Cloning of the *A. hydrophilia* CyaB gene in the PVP16 vector

The plasmid pTRC99ACyaB (gift from A. Danchin and A. Sekowska AMAbiotics SAS, Genopole Campus 1 - Genavenir 8, 91030 EVRY Cedex, France), containing untagged CyaB, was used as starting material. The *A. hydrophila* CyaB gene (Sismeiro *et al.* 1998) was amplified by polymerization chain reaction and inserted in the PVP16 vector (Song *et al.* 2008). To confirm the insertion of the gene, the plasmid was sequenced. The two primers that were used to amplify the gene are: CyaB Forward: 5′-TTGGCGCGCCCAGAAAACCTGTACTTCCAGTCCATGTCATCACAACACTTTCAGG-3′ and CyaB Reverse: 5′-TTACTAGTTCATGAGGGCACTTTCTCTGAG-3′. The ATG underlined in the sequence of CyaB Forward corresponds to the codon that encoded the first methionine in the protein sequence.

### Overexpression and purification of recombinant *A. hydrophila* CyaB

BL21DE3 were transformed with PVP16CyaB Vector and the expression of the protein was induced by using IPTG. This vector allows the expression of 8-histidine-tagged maltose binding protein (8HIS-MBP) fusion protein. After a first purification on a nickel column (1 mL HisTrap™ HP column (GE Healthcare) [1]), the tag was removed by Tobacco Etch Virus digestion and the purity was checked using Coomassie blue staining after SDS-PAGE.

### Purification of PPPase activity from pig brain

PPPase was purified from about 500 g of pig brain. All the purification steps were carried out at 4°C. The tissues were homogenized in Tris-HCl buffer (30 mM Tris-HCl, 50 mM, NaCl, 0.1 mM EDTA, 1.5 mM DTT, pH 7.4). The homogenate was centrifuged at 20,000×g for 20 minutes. The supernatant was brought to 80% saturation in ammonium sulfate and kept under constant stirring for 1 h. After centrifugation at 27,000×g for 30 minutes, the pellet was suspended in buffer A (20 mM Tris-HCl, 1 mM DTT, pH 7.4) and 5 ml aliquots were placed on a Sephadex G 75 column (3×50 cm, GE Healthcare Life Sciences) equilibrated with buffer A. The proteins were eluted with the same buffer at a flow rate of 2 mL/min and all the fractions with high PPPase activity were pooled. The pool was loaded on a DEAE-Sephacel anion exchange column (1.4×24 cm, GE Healthcare Life Sciences) equilibrated with buffer A. The proteins were eluted with an exponential concave NaCl gradient from 0 to 0.4 M in buffer A. Fractions with highest PPPase activity were pooled and centrifuged in Amicon ultra-15 centrifugal filter (Ultracel-10 membrane) units (Merck Millipore SA/NV, Overijse, Belgium). The retained proteins were dissolved in buffer B containing 20 mM Tris-HCl and 50 mM NaCl. 25 mg of protein were loaded on a Mono Q column (GE Healthcare Life Sciences) coupled to a Fast Protein Liquid Chromatography apparatus (ÄKTApurifier, Amersham) and elution was made with buffer B using a linear NaCl gradient from 50 to 500 mM. The fraction with the highest PPPase activity was pooled and DTT was added to reach a concentration of 1 mM. The proteins were then separated by non-denaturing polyacrylamide gel electrophoresis. The band with PPPase activity was cut and analyzed by liquid chromatography- mass spectrometry as described above for *E. coli* PPPase activity.

### Detection of PPP_i_ using anion exchange column and electrochemical detection

Ion chromatography coupled with a conductivity detector was used to detect the tripolyphosphate anion. The chromatograph (Dionex ICS-2000) was equipped with an Ion Pac AS 16 2×250 mm column, an Ion Pac AG16 2×50 mm guard column and an ARS 300 suppressor. The KOH gradient necessary for ion separation was generated by an EluGen EGC-KOH cartridge. The gradient profile was the following: start at 20 mM KOH, linear ramp to 45 mM for 4 minutes, step at this level during one minute to fully resolve the tri-meta phosphate from pyrophosphate followed by a second linear ramp up to 70 mM KOH during 3 minutes to elute highly charged polyphosphate ions. The ions were detected by an integrated Dionex DS6 conductivity detector after eluent suppression. The injection volume was 25 µL and the flow rate was 0.36 mL/min.

### Development of a method for PPP_i_ determination by capillary electrophoresis (CE)

CE experiments were carried out on a HP^3D^CE system (Agilent Technologies, Waldbronn, Germany) equipped with an incorporated diode array detector and a temperature control system (15–60°C±0.1°C). A CE Chemstation (Hewlett-Packard, Palo Alto, CA, USA) was used for instrument control, data acquisition and data handling. Fused-silica capillaries were provided by ThermoSeparation Products (San Jose, CA, USA).

Electrophoretic separations were carried out with uncoated fused-silica capillaries having 50 µm internal diameter and 70 cm length (61.5 cm to the detector). At the beginning of each working day, the capillary was washed with 1 N NaOH, water and the background electrolyte (BGE) for 10 min. Before each injection, the capillary was washed successively with water, for 1 min, 1 N NaOH for 3 min, water for 1 min and then equilibrated with the BGE for 5 min. The applied voltage was 12 kV in the negative polarity mode and UV detection was set at 260 nm. Injections were made by applying a voltage of −10 kV for a period of 12 s and the capillary was thermostated at 30°C. The composition of the BGE was optimized according to sensitivity and selectivity: the PPP_i_ peak (N: 233.000) was well separated from PP_i_ and P_i_. The BGE used for electrophoretic experiments was made up of 3 mM sodium molybdate in 200 mM malonate buffer adjusted to pH 3: acetonitrile (ACN) (80∶20; v/v). The determination of PPP_i_ is based on the in-capillary formation of anionic polyoxomolybdate complex in the presence of ACN, as already described for phosphonate, P_i_ and PP_i_ [40]. *E. coli* supernatants were injected into the CE system after fractionation on a G-25 column.

### Preparation of samples for PPP_i_ determination

*E. coli* (MG1655 strain) were grown overnight in 50 ml Luria Bertani medium and centrifuged (10 min at 8000×g). The pellet was suspended in 500 µL 20 % TCA and centrifuged for 5 minutes at 13,000×g. The TCA suspension was then extracted 10 times with 3 volumes of diethyl ether. The sample was injected onto a 3.9 mL G-25 column and fractions of 500 µl were collected in 10 mM Hepes pH 7. In the control samples, 83 µL of 72% TCA were added to 417 µL 10 mM PPP_i_ and treated as above.

## Supporting Information

Table S1 **Purification of tripolyphosphatase activity from** ***E. coli*** **soluble fraction.**
(DOCX)

Figure S1 **Purification of the GST-ygiF fusion protein.** (Lane 1, molecular mass weight markers; Lane 2, bacterial supernatant; lane 3, purified protein after the GST column [2]).
(TIFF)

Figure S2 **PPPase activity as a function of pH in the supernatants of MG1655 (WT) and JW3026-2 (YgiF KO) strains at 37°C** (0.5 mM PPP_i_, 5 mM Mg^2+^).
(TIFF)

Figure S3 **PPPase activity in the supernatants of MG1655 (WT) and DPPK-PPX (CF5802) and DygiF (JW3026-2) strains.** The supernatants were incubated for 20 min at 50°C (5 mM PPP_i_, 5 mM Mg^2+^ in TAPS buffer at pH 9.5). (Means ± SD, n = 3).
(TIFF)

Figure S4 **PPase (blue) and PPPase (red) activities as a function of growth in** ***E. coli*** **.** Growth was measured by following the absorbance at 600 nm (black curve).
(TIFF)

Figure S5 **Sequence alignment of** ***Drosophila*** **, pig and human prune.** The sequence alignment was made using CLC Sequence Viewer 6 (CLC bio A/S, 8200 Aarhus, Denmark).
(TIFF)
