# Supplementary material for: High Inorganic Triphosphatase Activities in Bacteria and Mammalian Cells: Identification of the Enzymes Involved
Source: PLoS One. 2012 Sep 12;7(9):e43879. doi: 10.1371/journal.pone.0043879 (PMC3440374; doi:10.1371/journal.pone.0043879)
Supplement: Table S1 — Purification of tripolyphosphatase activity from E. coli soluble fraction. (DOCX) [file pone.0043879.s001.docx]

**Table S1**

Purification of tripolyphosphatase activity from *E. coli* soluble fraction.

| Step | Fraction | Protein (mg) | Activity (50 °C)  (µmol/min) | Specific activity  (µmol/mg.min) | Step purification factor | Total purification factor |
| --- | --- | --- | --- | --- | --- | --- |
| Homogenate supernatant  Ammonium sulfate precipitation  MonoQ  Sephadex  G200 | S1  C80  F1  F2 | 428  163  4.69  3.09 | 77  160  24  3 | 0.18  0.98  5.1  7.5 | 1  5,45  5,17  1,47 | 1  5.5  28  41 |
