# Supplementary figures and images for: High Inorganic Triphosphatase Activities in Bacteria and Mammalian Cells: Identification of the Enzymes Involved
Source: PLoS One. 2012 Sep 12;7(9):e43879. doi: 10.1371/journal.pone.0043879 (PMC3440374; doi:10.1371/journal.pone.0043879)

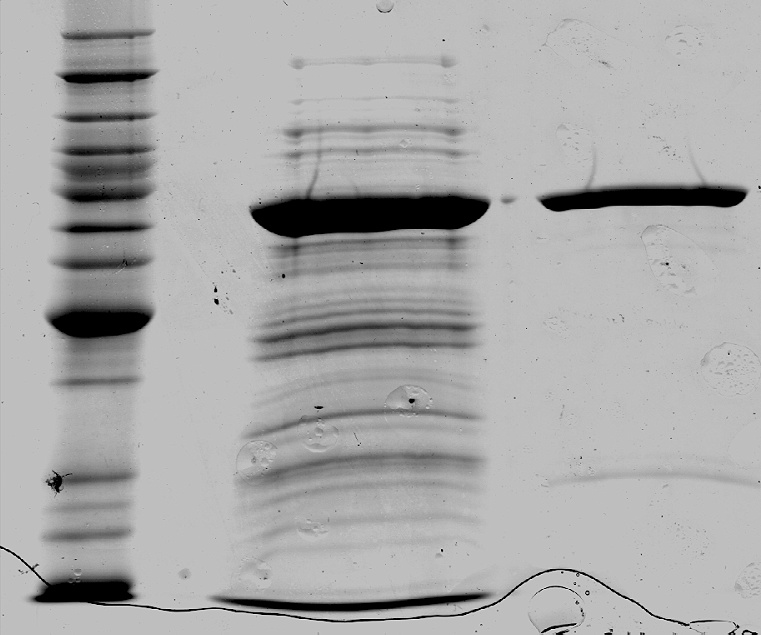

Supplement: Figure S1 — Purification of the GST-ygiF fusion protein. (Lane 1, molecular mass weight markers; Lane 2, bacterial supernatant; lane 3, purified protein after the GST column [2]). (TIFF) [file pone.0043879.s002.tiff]

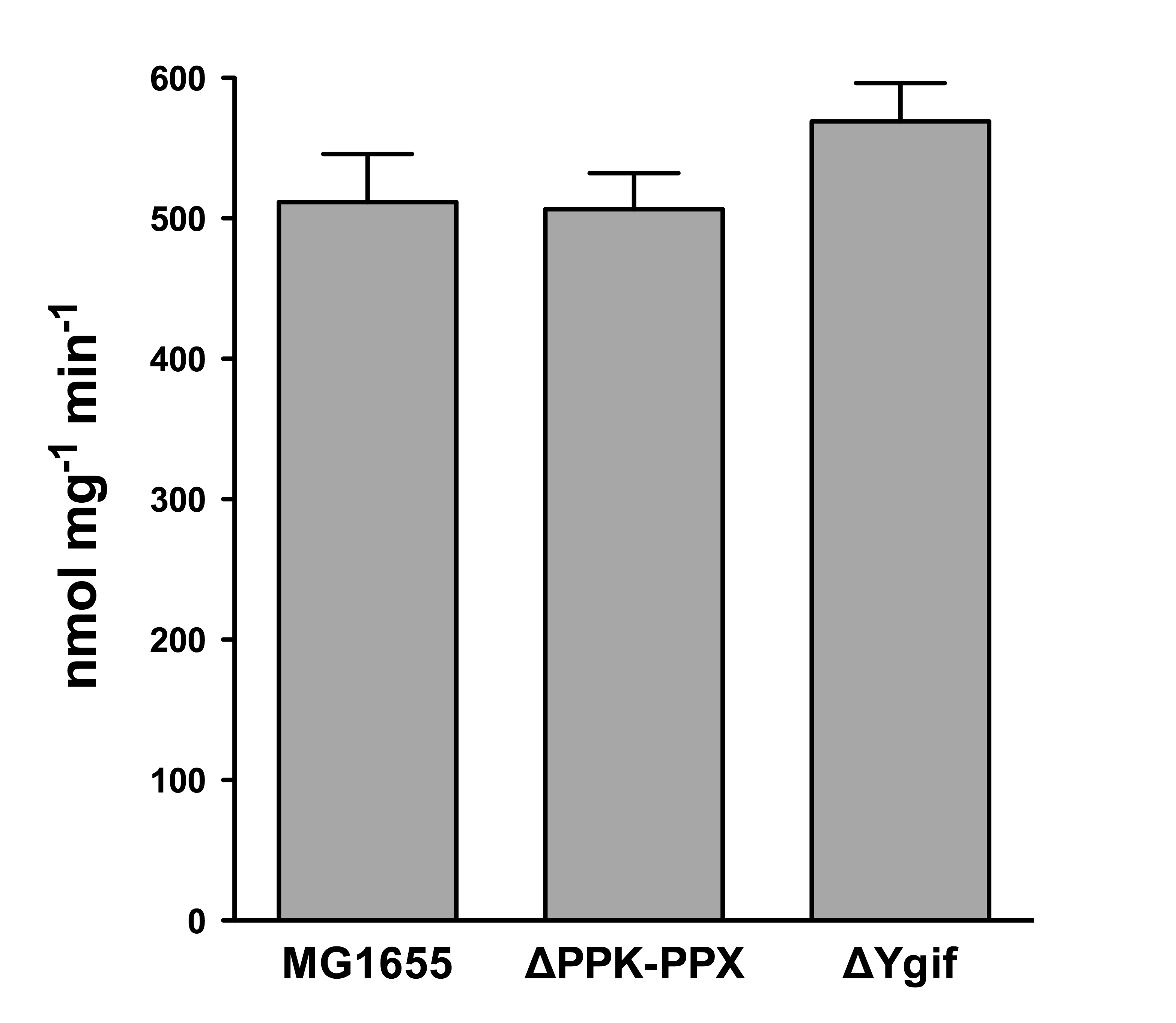

Supplement: Figure S3 — PPPase activity in the supernatants of MG1655 (WT) and DPPK-PPX (CF5802) and DygiF (JW3026-2) strains. The supernatants were incubated for 20 min at 50°C (5 mM PPPi, 5 mM Mg2+ in TAPS buffer at pH 9.5). (Means ± SD, n = 3). (TIFF) [file pone.0043879.s004.tiff]

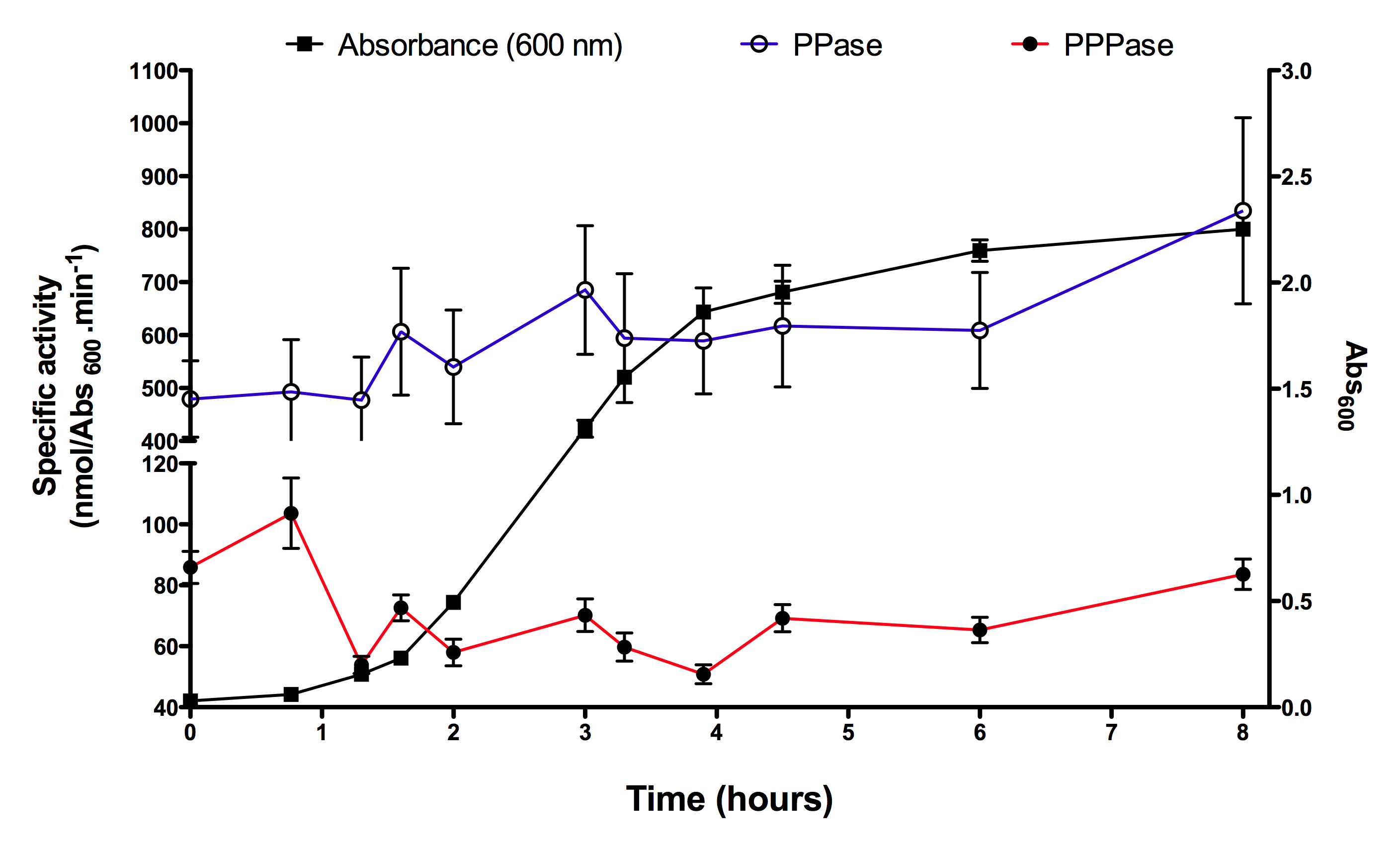

Supplement: Figure S4 — PPase (blue) and PPPase (red) activities as a function of growth in E. coli . Growth was measured by following the absorbance at 600 nm (black curve). (TIFF) [file pone.0043879.s005.tiff]
